# Screening Test for At-Risk Drinking: Development of New Abbreviated Version of Alcohol Use Disorder Identification Test for Young and Middle-Aged Adults

**DOI:** 10.1155/2018/2306587

**Published:** 2018-05-17

**Authors:** Jae Hee Lee, Koo Young Jung, Yoon Hee Choi

**Affiliations:** ^1^Department of Emergency Medicine, Ewha Womans University Mokdong Hospital, Seoul, Republic of Korea; ^2^Department of Emergency Medicine, College of Medicine, Ewha Womans University, Seoul, Republic of Korea

## Abstract

Several abbreviated versions of the Alcohol Use Disorders Identification Test (AUDIT) have been developed for use in high-volume clinical situations such as emergency departments. In this study, we developed a new abbreviated version of AUDIT called the Screening Tool for At-risk Drinking (STAD) for young and middle-aged adults, consisting of two questions that reflect the structure of the AUDIT questionnaire using data from the Korea National Health and Nutrition Examination Survey (KNHANES). To derive the abbreviated test considering AUDIT item structure, we performed confirmatory factor analysis on the 10 AUDIT questions in the Korea National Health and Nutrition Examination Survey (KNHANES) IV. To validate the new abbreviated test, we analyzed the sensitivity, specificity, and area under the receiver operating characteristic curve (AUROC) on the KNHANES V-VI except for the KNHANES VI-2. Based on the two-factor structure of AUDIT, question (Q) 3 and Q7 were finally selected for STAD. In validation, AUROC was significantly wider for STAD than for AUDIT-QF, which has the same number of questions. There was no significant difference between AUDIT-C, consisting of three questions, and STAD. It can be used as a simple and reliable screening test in clinical settings.

## 1. Introduction

The Alcohol Use Disorder Identification Test (AUDIT) developed by the World Health Organization (WHO) in 1989 is now the most widely used test method to screen for at-risk drinking [1]. The development of AUDIT was based on multinational studies starting in 1987. The developers collected data consisting of 150 questions and sorted them into conceptual groups. Four groups were selected through correlation analysis and factor analysis of intrascale reliability and daily mean alcohol consumption. Then, the developers selected two or three questions per group, considering the weighted mean item-to-total correlation coefficient in each of the four groups; from these candidates, they assembled AUDIT, consisting of 10 questions [1–3]. AUDIT questions 1 to 3 (Q1–Q3) are related to the amount and frequency of drinking, questions 4 to 6 (Q4–Q6) are related to alcohol dependence symptoms, and questions 7 to 10 (Q7–Q10) are related to alcohol-related problem.

In United States, alcohol abuse is one of the main public health problems among young adults [4, 5]. Despite the brief intervention, young adults still have alcohol drinking problems [6]. According to the data from Korea National Health and Nutrition Examination (KNHANES) conducted from 2007 to 2015, the risk drinking group was 41.2% in the 20s, 35.5% in the 30s, 33.4% in the 40s, and 29.0% in the 50s. However, it decreases in 60s as 21.5%, in the 70s as 13.7%, and in the 80s and older as 5.9% [7]. Screening and brief intervention for at-risk drinking among young and middle-aged adults are important. National Institute on Alcohol Abuse and Alcoholism (NIAAA) proposed several clinical situations as key opportunities for screening at-risk drinking. It includes emergency department or urgent care center, prescribing a medicine that interacts with alcohol, and so on [8]. Many studies have shown that brief intervention about alcohol drinking in emergency department is effective [9–12]. Emergency department is an important clinical place for screening at-risk drinking and conducting brief intervention. However, it is not practically feasible to carry out all 10 AUDIT questions in many outpatient clinics or crowded emergency department. Therefore, research has been conducted to develop and test abbreviated versions of AUDIT as simple screening instruments that can be administered quickly [13–20].

For AUDIT-Q3 alone, AUDIT-QF, and AUDIT-C, items were selected based on past researches [19, 21], whereas AUDIT-4 and AUDIT-PC extracted the items using logistic regression [14, 22]. FAST used a method to compare the sensitivity and specificity of the combinations of the questions after the questions were analyzed by principal component analysis [23]. However, previous abbreviated tests have a limitation in that they do not properly reflect the AUDIT survey structure. As the number of questions increases, the accuracy will increase. However, since it is not easy to conduct the tests, we tried to develop a two-question abbreviated test to replace AUDIT-C, which is widely used today.

At the time the AUDIT was developed, it consisted of three domains: Q1 to Q3, alcohol consumption factors; Q4 to Q6, alcohol dependence factors; and Q7 to Q10, harmful alcohol use. However principal component analysis and factor analysis in most studies supported a two-factor structure consisting of Q1–Q3 and Q4–Q10 [24–37]. This means that the structure of AUDIT in 3 domains can be divided into two major categories. In this study, we tried to develop new abbreviated test designated for young and middle-aged adults, the Screening Tool for At-risk Drinking (STAD), consisting of two questions that reflect the structure of the AUDIT questionnaire, using data from KNHANES, which are representative of the Republic of Korea.

## 2. Material and Methods

### 2.1. Setting and Data Collection

Data from the fourth KNHANES (KNHANES IV, 2007–2009) were used to extract questions for STAD. Data were obtained by requesting primitive data through the National Health and Nutrition Survey homepage of the Korea Centers for Disease Control and Prevention [38]. The sample design of the KNHANES IV used the 2005 Population and Housing Census data as a sampling framework. The first extraction unit was the village, the second extraction unit was the population housing survey unit, and the third extraction unit was the household. Socioeconomic location indexes and weights as well as a common variable survey, a health questionnaire, an examination survey, a nutrition survey, and other measures were conducted for selected subjects in the survey area. The health questionnaire contains 12 alcohol-related questions, including 10 questions based on the AUDIT 10 questions and two questions about drinking experience. For the validation of the newly developed abbreviated test, we used the alcohol-related contents of the fifth KNHANES (KNHANES V, 2010–2012) and the sixth KNHANES (KNHANES VI, 2013–2015). The alcohol-related contents were the same except in the second year of the KNHANES VI (KNHANES VI-2, 2014). Therefore, the data from the KNHANES VI-2 were excluded.

### 2.2. Outcome Measure

Among the subjects between 19 and 64 years old in the KNHANES IV, we applied the cut-off values (8 for males, 7 for females) for at-risk drinking proposed by WHO and divided the sample into normal and at-risk drinking groups [39]. To derive an abbreviated test considering the item structure of AUDIT, we performed confirmatory factor analysis on AUDIT 10 questions of the KNHANES IV. Based on the previous literature, we adopted a two-factor structure consisting of Q1–Q3 and Q4–Q10 [24–36]. In determining the number of questions in a new abbreviated test, the area under the receiver operating characteristic curve (AUROC) increases when the number of questions increases, but the function of the abbreviated test is inferior. Considering that AUDIT-C, the most widely used existing abbreviated test, is composed of 3 questions, this study attempts to develop a simpler two-question abbreviated test. Based on the score distribution and confirmatory factor analysis results of AUDIT 10 questions, one question was selected for each factor, and a new abbreviated test was derived. First, considering factor loading in the literature, we chose the questions based on the AUROC, sensitivity and specificity of each combination of questions. For the validation of the new abbreviated test, its sensitivity, specificity, and AUROC were analyzed on the KNHANES V-VI except for KNHANES VI-2.

### 2.3. Statistical Analysis

The fit of factor structure was assessed using chi-squared analysis, the Bentler comparative fit index (CFI), the root mean square error of approximation (RMSEA), and root mean square residual (RMSR). The cut-off values were *X*^2^, *P* > 0.05 [40]; CFI > 0.90 [40]; RMSEA ≤ 0.08 [41]; and RMSR ≤ 0.08 [42]. For the validation of the new abbreviated test, the analysis was performed using the program SAS CALIS (SAS Institute, Cary, NC). The analysis programs PASW 18 and MedCalc Statistical Software version 17.2 (MedCalc Software bvba, Ostend, Belgium) were used.

## 3. Results

The KNHANES IV was conducted from 2007 to 2009 among 14,334 people aged between 19 and 64 years, including 6,278 men (43.8%) and 8,056 women (56.2%) with an average age of 42.3 years. Of these, 34.6% were in the at-risk drinking group (defined by a total AUDIT score of more than 8 for men, 7 for women). The KNHANES V~VI except VI-2 was conducted from 2010 to 2013 and 2015 among 23,992 people aged between 19 and 64 years, including 10,450 men (43.6%) and 13,542 women (56.4%) with an average age of 43.5 years.

Based on our review of the previous literature on the factor structure of AUDIT, we adopted a two-factor structure consisting of Q1–Q3 and Q4–Q10. The chi-squared analysis and the CFI, RMSEA, and RMSR values that determine the fit of the factor structure were statistically significant. The items with the greatest factor loadings were Q3 in the first factor and Q7 and Q8 in the second factor (Table 1). After selecting Q3 from the first factor and Q7 and Q8 from the second, we compared AUROC for the combinations of Q3, Q7 and Q3, Q8. The AUROC for the combination of Q3 and Q7 was greater; therefore, Q3 and Q7 were ultimately selected (Table 2).

The minimum total AUDIT score for the at-risk drinking group was defined as 8 for males and 7 for females. The cut-off values derived in the development of STAD were 3 for males and 2 for females. As a result, the sensitivity/specificity were 86.65/96.27 and 97.06/88.46, respectively, for male and female (Table 3). We compared the utility of the existing abbreviated tests AUDIT-QF and AUDIT-C with that of the new abbreviated test, STAD. AUROC was significantly wider for STAD than for AUDIT-QF, which has the same number of questions. There was no significant difference between AUDIT-C, consisting of three questions, and STAD (Table 4).

## 4. Discussion

AUDIT consists of 3 parts: questions 1 to 3 (Q1–Q3) are related to the amount and frequency of drinking, alcohol consumption; questions 4 to 6 (Q4–Q6) are related to alcohol dependence symptoms; questions 7 to 10 (Q7–Q10) are related to alcohol-related problem. In the case of the existing abbreviated versions of AUDIT, the development was carried out in various ways, using the previous research [19, 21] and logistic regression analysis [14, 22] to select test items. In another abbreviated version of AUDIT, a principal component analysis was performed to select one question, and the remaining questions were combined to select the one with the highest sensitivity and specificity [23]. AUDIT-C, the most widely used abbreviated version of AUDIT [43], is limited to three questions about the consumption of alcohol and does not reflect the structure of the AUDIT 10 questions. As the number of questions increases, the accuracy of the abbreviated test will increase, but it becomes more difficult to conduct the abbreviated test. Therefore, we wanted to develop two-question abbreviated tests that could replace AUDIT-C, which is widely used at present. In clinical practice, many of the existing tests are abbreviated. For example, 12 items were selected among 36 items by regression analysis in the process of reducing the 36-Item Short-Form Health Survey (SF-36) to the 12-Item Short-Form Health Survey (SF-12) [44]. Another test, the CES-D scale (the Center for Epidemiologic Studies Depression Scale) for diagnosing depression, classifies many questions that have been verified as indicators of depression in previous studies as a set; 20 items were selected and classified under 4 factors [45]. Subsequently, 10 items that strongly correlated with the 4 factors in factor analysis were selected [46]. In this study, we used regression analysis to select two questions out of the 10 AUDIT questions; however, since the diagnostic criterion for at-risk drinking was the total score of the AUDIT 10 questions, no questions could be removed and the problem could not be addressed. Therefore, we decided to select the questions considering the factor structure of AUDIT. In the previous literature, test structures with one to three factors have been proposed. At the time when the AUDIT questions were developed, they consisted of three domains (Q1 to Q3, alcohol consumption factors; Q4 to Q6, alcohol dependence factors; and Q7 to Q10, harmful alcohol use factors). In the majority of studies, principal component analysis and factor analysis have supported a two-factor structure consisting of Q1–Q3 and Q4–Q10 [24–37]. In this study, we tried to construct a new abbreviated version of AUDIT with two questions by selecting one question from each of the two factors of AUDIT. For the first factor, we selected Q3, which had the highest factor loading, based on the results of the factor analysis and previous research [24–31]. For the second factor, Q7 and Q8, which had the highest factor loadings, were selected first. Subsequently, the combinations of Q3, Q7 and Q3, Q8 were compared in terms of AUROC, sensitivity, and specificity. Based on the results of the comparison, we selected Q3 and Q7 for the new abbreviated test, STAD. In the validation of the newly developed tests on the KNHANES V-VI except for the KNHANES VI-2, there is no statistically significant difference in AUROC between AUDIT-C and STAD. In other words, the number of questions was reduced, but the reliability of the test was unaffected. This means that STAD is superior in utility and addresses the structural limitations of AUDIT-QF and AUDIT-C, which are currently in widespread use.

This study uses the data of the KNHANES, the representative health indicator of the Republic of Korea, to develop a new abbreviated version of AUDIT based on the factor structure. We have presented effective means to screen for at-risk drinking in crowded environments such as emergency departments and primary care rooms. Drinking is not merely a subject of personal preference but a problem that needs to be assessed from a national perspective. The severe consequences of excessive alcohol consumption are already well known, and there is no question that systematic interventions should be made. It is important to understand the actual state of drinking in the Republic of Korea. Proper intervention must be applied to the at-risk population, and we facilitate such targeted intervention by developing a screening test for at-risk drinking. In addition, we believe our screening tool will facilitate research into the problem of alcohol abuse.

## 5. Limitations

The limitation of this study is that the questionnaire related to alcohol in the KNHANES did not record the exact amount of alcohol consumption or frequency of intake. If the criteria recommended by the National Institute on Alcohol Abuse and Alcoholism were used in the diagnosis of at-risk drinking, the study would be more reliable and easier to compare with other studies. Furthermore this study is based on data from public health research, it needed further studies to validate its usefulness in clinical settings.

## 6. Conclusions

This study developed efficient and accurate new abbreviated tests using national data. Specifically, we developed STAD, consisting of Q3, Q7 of AUDIT. We consider STAD to be a useful test reflecting the structure of AUDIT and the characteristics of each population group. It is expected that this test can replace previous abbreviated tests and facilitate screening for at-risk drinking.

## Figures and Tables

**Table 1:** Factor loadings for confirmatory factor analysis of AUDIT.

	Two-factor solution			
	Factor 1	Factor 2	Model fit	
Q1	0.78	0		
Q2	0.85	0		
Q3	0.96	0		
Q4	0	0.76	*X* ^2^ (df)	1345.01 (32), *P* < 0.0001
Q5	0	0.73	CFI	0.9705
Q6	0	0.38	RMSEA	0.07
Q7	0	0.77	RMSR	0.03
Q8	0	0.77		
Q9	0	0.37		
Q10	0	0.61		

*Notes*. AUDIT = Alcohol Use Disorders Identification Test; CFI = comparative fit index; RMSEA = root mean square error of approximation; RMSR = root mean square residual.

**Table 2:** Comparison of AUROCs between Q3 + Q7 and Q3 + Q8 in KNHANES IV.

	Question combination	AUROC	95% CI	*P* value
Male	Q3 + Q7	0.97	0.964–0.973	<0.0001_ _^*∗*^
	Q3 + Q8	0.964	0.959–0.969	
Female	Q3 + Q7	0.976	0.973–0.980	<0.0001_ _^*∗*^
	Q3 + Q8	0.967	0.963–0.971	

*Notes*. AUROC = area under the receiver operating characteristic curve; KNHANES IV = the fourth Korea National Health and Nutrition Examination; CI = confidence interval. *∗* means the significantly highest value than other groups.

**Table 3:** Cut-off values, sensitivities, and specificities of STAD.

	Sensitivity, %		Specificity, %		+LR (95% CI)		−LR (95% CI)	
STAD								
Male								
≥2	97.45	(96.8–98.0)	77.24	(75.5–78.9)	4.28	(4.0–4.6)	0.033	(0.03–0.04)
≥3_ _^*∗*^	86.48	(85.3–87.6)	95.63	(94.8–96.4)	19.8	(16.5–23.8)	0.14	(0.1–0.2)
≥4	63.21	(61.5–64.9)	99.56	(99.2–99.8)	143.4	(79.5–258.8)	0.37	(0.4–0.4)
Female								
≥1	99.42	(98.9–99.7)	70.16	(69.0–71.3)	3.33	(3.2–3.5)	0.008	(0.004–0.02)
≥2_ _^*∗*^	97.45	(96.5–98.2)	88.01	(87.2–88.8)	8.12	(7.6–8.7)	0.029	(0.02–0.04)
≥3	79.84	(77.6–81.9)	98.07	(97.7–98.4)	41.31	(34.6–49.4)	0.21	(0.2–0.2)

*Notes*. STAD = screening test for at-risk drinking; LR = likelihood ratio; CI = confidence interval. *∗* means recommended cut-off values of STAD.

**Table 4:** Comparison of AUROCs between STAD and abbreviated version of the AUDIT in KNHANES V-VI.

	Question combination	AUROC	Test name	AUROC	*P* value
Male	STAD (Q3 + Q7)	0.97	AUDIT-QF	0.96	<0.0001_ _^*∗*^
			AUDIT-C	0.973	0.0965
Female	STAD (Q3 + Q7)	0.976	AUDIT-QF	0.966	<0.0001_ _^*∗*^
			AUDIT-C	0.978	0.151

*Notes*. AUROC = area under the receiver operating characteristic curve; STAD = screening test for at-risk drinking; AUDIT = Alcohol Use Disorders Identification Test; KNANES V-VI = the fifth and sixth Korea National Health and Nutrition Examination. *∗* means the significantly highest value than other groups.

## Data Availability

The data used to support the findings of this study are available from the corresponding author upon request.
